# A vascularized 3D model of the human pancreatic islet for *ex vivo* study of immune cell-islet interaction

**DOI:** 10.1088/1758-5090/ad17d0

**Published:** 2024-01-11

**Authors:** R Hugh F Bender, Benjamen T O’Donnell, Bhupinder Shergill, Brittany Q Pham, Sima Tahmouresie, Celeste N Sanchez, Damie J Juat, Michaela M S Hatch, Venktesh S Shirure, Matthew Wortham, Kim-Vy Nguyen-Ngoc, Yesl Jun, Roberto Gaetani, Karen L Christman, Luc Teyton, Steven C George, Maike Sander, Christopher C W Hughes

**Affiliations:** 1 Department of Molecular Biology & Biochemistry, University of California, Irvine, CA, United States of America; 2 Department of Biomedical Engineering, University of California, Davis, CA, United States of America; 3 Pediatric Diabetes Research Center, Department of Pediatrics, University of California, San Diego, CA, United States of America; 4 Department of Cellular & Molecular Medicine, University of California, San Diego, CA, United States of America; 5 Department of Bioengineering, University of California, San Diego, CA, United States of America; 6 Department of Molecular Medicine, Sapienza University of Rome, Rome, Italy; 7 Department of Immunology & Microbiology, The Scripps Research Institute, San Diego, CA, United States of America; 8 Department of Biomedical Engineering, University of California, Irvine, CA, United States of America

**Keywords:** microphysiological systems, organ-on-a-chip, diabetes, islet biology, glucose-stimulated insulin secretion

## Abstract

Insulin is an essential regulator of blood glucose homeostasis that is produced exclusively by *β* cells within the pancreatic islets of healthy individuals. In those affected by diabetes, immune inflammation, damage, and destruction of islet *β* cells leads to insulin deficiency and hyperglycemia. Current efforts to understand the mechanisms underlying *β* cell damage in diabetes rely on *in vitro*-cultured cadaveric islets. However, isolation of these islets involves removal of crucial matrix and vasculature that supports islets in the intact pancreas. Unsurprisingly, these islets demonstrate reduced functionality over time in standard culture conditions, thereby limiting their value for understanding native islet biology. Leveraging a novel, vascularized micro-organ (VMO) approach, we have recapitulated elements of the native pancreas by incorporating isolated human islets within a three-dimensional matrix nourished by living, perfusable blood vessels. Importantly, these islets show long-term viability and maintain robust glucose-stimulated insulin responses. Furthermore, vessel-mediated delivery of immune cells to these tissues provides a model to assess islet-immune cell interactions and subsequent islet killing—key steps in type 1 diabetes pathogenesis. Together, these results establish the islet-VMO as a novel, *ex vivo* platform for studying human islet biology in both health and disease.

## 1. Introduction

Diabetes affects over 34 million individuals in the U.S. and is broadly grouped as type 1 (T1D), which is characterized by early (Juvenile) onset, and type 2 (T2D), a complex metabolic disorder. Both types are characterized by blood glucose dysregulation and hyperglycemia [1]. T1D is caused by deficiency of the hormone insulin, which regulates blood glucose homeostasis and is produced exclusively by *β* cells residing in pancreatic islets. During T1D pathogenesis, islet *β* cells are destroyed, leading to insulin deficiency and subsequent hyperglycemia [2]. Later stages of T1D are characterized by T-Cell invasion of islets and T-Cell mediated destruction of *β* cells. Numerous theories have been put forward addressing mechanisms behind T-Cell activation and recruitment, although no definitive mechanism has been described. It is well accepted that T-Cell activation and recruitment is a result of multifactorial interactions between inherited stimuli, such as associations with HLA-DR [3] and HLA-DQ [4, 5], and environmental stimuli, such as chronic low-grade inflammation [6]. In contrast, T2D is characterized by insulin resistance in peripheral tissues and relative insulin deficiency, caused by chronic damage to *β* cells [7], which may result from cytokine-mediated activation of tissue-resident macrophages driving islet inflammation [8]. Although the exact mechanisms of diabetes pathogenesis remain unclear, inflammation and/or autoimmune reactivity against *β* cells are key drivers in both forms of diabetes [9, 10]. To date, most diabetes studies have relied on mouse models to dissect the drivers of disease pathogenesis, yet these models neglect important differences in islet structure, function, and immunology between human and mouse [11, 12]. Thus, there is increased emphasis on developing better *in vitro* tools that utilize human islets to study diabetes.

In the native pancreas, human islets reside within highly vascularized islet niches that ensure proper islet function. During development, endocrine precursor cells co-develop with endothelial cells (EC) such that blood vessels surround and penetrate mature islets in the adult pancreas, localizing next to endocrine cells to enable sensing of blood glucose levels and rapid transport of signaling hormones [13–16]. The surrounding extracellular matrix (ECM), which includes a laminin-rich basement membrane, supports integrin binding and signaling cues that drive *β* cell proliferation, and insulin expression and release [17, 18].

Standard islet isolation procedures physically disrupt and remove both blood vessels and ECM, causing structural damage, reduced glucose responsiveness, and death of a proportion of islets [19, 20]. Moreover, these isolated islets maintain their function for only a few days [19, 20], underscoring the need for new approaches that better maintain isolated human islets. Pancreatic islets have been characterized with high intra- and inter-donor heterogeneity in size, cell composition, and glucose responsiveness that necessitates large study populations and complicated *in vitro* experimental designs to recapture islets for further analysis [21]. There is thus a need for development of *in vitro* techniques that allow for non-destructive analysis of islets. This along with the role of the microenvironment in promoting islet health *in vivo* suggests biomimetic models could provide the necessary cues for preserving islet viability and function during prolonged culture.

Previously, we developed a microfluidic vascularized micro-organ (VMO) platform designed to grow perfusable human blood vessels within a three-dimensional (3D) hydrogel matrix that mimics the *in vivo* environment [22–24]. The resulting capillary-like vessels deliver nutrients directly to and remove waste from the surrounding tissue, just as in the body [25, 26]. *In vivo*, blood vessels also carry immune cells, including the cytotoxic T cells thought to destroy β cells during T1D pathogenesis. Here, we present a VMO platform that incorporates human cadaveric islets within a 3D vascularized tissue—the islet-VMO. The platform models the native islet niche, with β cells receiving glucose via the surrounding vasculature and releasing insulin in response.

## 2. Research design and methods

**Figure 1.**
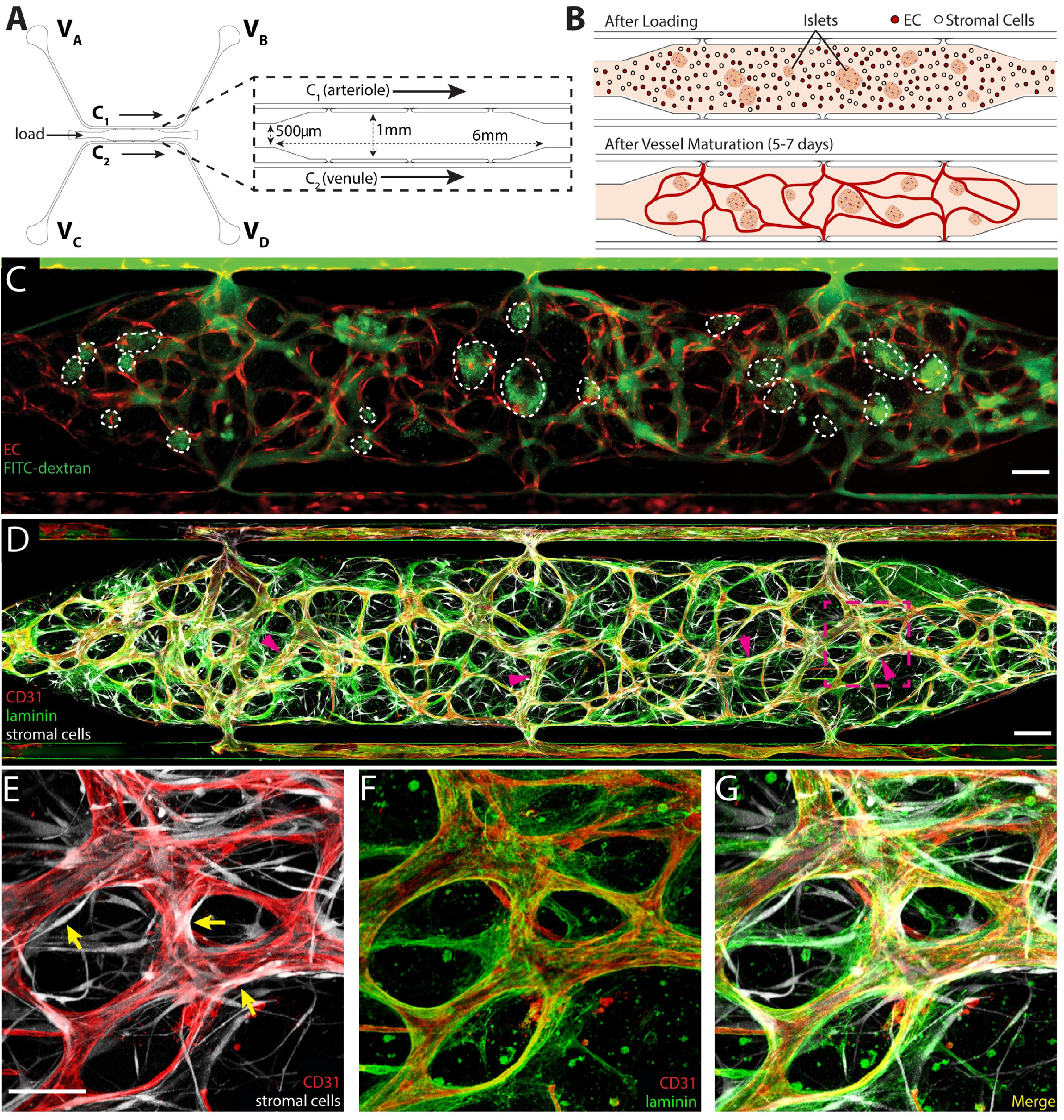
Generating blood vessels in an islet-specialized vascularized micro-organ (islet-VMO). (A) A microfluidic design for generating vascularized islet tissues comprises a central, enlarged cell chamber (inset) flanked by two channels, C_1_ (acting as an arteriole) and C_2_ (venule). Reservoirs at the end of each channel (V_A_–V_D_) are filled with varying heights of medium such that medium is driven by hydrostatic pressure in a net direction from V_A_, through the tissue chamber, and to V_D_. (B) To generate vascularized tissues, islets, EC, and stromal cells are loaded together in a fibrin hydrogel through the loading tunnel (labeled in A). Fluid flow is driven across the cell chamber to activate vessel formation in the central chamber with development of intact vessel networks by 5–7 d post-loading. (C) Upon maturation, blood vessels (fluorophore-transduced ECs, red) carry 70 kDa FITC-dextran (green) with minimal leak. (D), (E), (G) Mature vessels are wrapped by stromal (pericyte) cells (magenta arrows; transduced stromal cells, white). (D), (F), (G) These stromal cells help remodel the hydrogel to generate basement membrane (laminin, green) around the CD31^+^ endothelium (red). Scale bar, 200 *μ*m, inset, 100 *μ*m.

***Platform fabrication.*** The islet-VMO microfluidic platform was fabricated using standard PDMS photolithography techniques as previously described [23]. Briefly, Silicon wafers were coated with 200 *μ*m thick SU-8 100 (Micro Chem Westborough, MA). Single mask photography was used to define the microchannels as detailed in figure 1(A). SU-8 coating was silanized with trichlorosilane (C_8_H_4_Cl_3_F1_3_Si) to generate a master mold. Devices were cast with polydimethylsiloxane (PDMS, Ellsworth Adhesives, Germantown WI) in the SU-8 master molds. PDMS devices were then demolded and plasma bonded to a 1 mm thick PDMS layer to create microfluidic channels.

***Islet isolation.*** Non-diabetic human cadaveric islets were provided by the NIDDK Integrated Islet Distribution Program or Prodo Laboratories Inc. (Aliso Viejo CA). Upon arrival, islets were washed in short-term islet medium (table S1) then stained with dithizone. Dithizone^+^ islets <200 *μ*m in diameter were hand-selected under a dissection microscope using a micro-ruled coverslip (Nexcelom Bioscience, Lawrence MA) then allowed to recover overnight in islet medium prior to subsequent studies.

***Islet-VMO loading.*** Islets were co-loaded (20–25 islets *μ*l^−1^ hydrogel) with 7 × 10^6^ cells ml^−1^ of endothelial colony-forming endothelial cells (ECFC-ECs, ‘EC’) and stromal cells (NHLFs, Lonza Bioscience, Rockville MD) in 8 mg ml^−1^ fibrin hydrogel clotted with 10 U ml^−1^ thrombin (Sigma-Aldrich, St. Louis MO). Islets were maintained within the islet-VMO for up to two weeks by gravity-driven flow of EGM-2 medium (Lonza Bioscience) through the vasculature [24, 26, 27]. The provisional fibrin gel is rapidly remodeled by the stromal cells into a complex, collagen-rich ECM [28].

***Vascular network quantification.*** Vessel networks were perfused with 50 *μ*g ml^−1^ 70 kDa FITC or rhodamine-dextran (Sigma-Aldrich) and imaged on a Nikon Ti-E Eclipse (Nikon Instruments Inc., Melville NY) inverted fluorescence microscope. Morphological measurements of perfused vessels were taken using FIJI imaging software [29] by tracing the mid-point of all vessels through the cell chamber (figure S1). Branch points were quantitated at the intersection of these mid-point lines. Lastly, vessel diameter was determined by measuring across each traced vessel at ∼50 *μ*m intervals.

***Immunofluorescence staining.*** Islets were fixed (4% paraformaldehyde), dehydrated (30% sucrose, >24 h), embedded in OCT mounting medium, and 10 *μ*m sections were collected. Sections were incubated with primary antibodies (table S2) overnight at 4 °C, then with species-appropriate secondary antibodies, and finally DAPI counterstained. Islet-VMO platforms were perfused with 4% paraformaldehyde (30 min, 25 °C), then flushed with DPBS. The bottom silicon membrane was removed prior to antibody staining (as described above). Confocal image stacks of sectioned and device-embedded islets were acquired on a Leica TCS SP8 confocal microscope.

***Static glucose-stimulated insulin secretion (GSIS).*** GSIS assays were performed to confirm islet function. Recovered islets were starved in low-glucose Krebs-Ringers Buffer (KRB, table S1) (1 h, 37 °C), then incubated in five groups of ten islets per well in either standard (2.8 mM glucose) KRB or high-glucose KRB (16.7 mM glucose) for 1 h at 37 °C. Supernatant was collected and frozen for subsequent insulin assay. Islets were also lysed in acid ethanol (4 °C) and total protein was collected for total insulin measurements. Insulin was measured by human insulin ELISA (Mercodia, Uppsala Sweden).

***Islet-VMO GSIS.*** To maintain stability of the vasculature, devices were perfused with M199 medium (‘Standard M199(+),’ table S1) instead of KRB media. Standard M199 basal media contains 5.5 mM glucose and was perfused through the device for one hour to establish a baseline insulin secretion. Islets were then stimulated for one hour by adding M199(+) containing 16.7 mM glucose (‘High-Glucose M199(+)’) to reservoir V_A_ (figure 1(A)), followed by Standard M199(+) for two hours to allow islets to return to baseline insulin secretion levels. M199(+) supplemented with 30 mM KCl was then perfused for one hour to assay for releasable insulin. Accumulated device flow-through was collected continuously at 10-minute intervals beginning thirty minutes prior to High-Glucose M199(+) stimulation. Glucose concentration in the flow-through was assessed using a Contour glucometer (Ascensia Diabetes Care, Parsippany NJ). Insulin was measured using an ultra-sensitive insulin ELISA assay (Mercodia). Insulin fold change was defined as insulin secretion divided by the average insulin secretion during the initial starvation period (−20–0 min).

***GSIS modeling.*** Mathematical modeling was performed in COMSOL Multiphysics (COMSOL, Inc., Burlington MA). 2D models of the islet-VMO were created in AutoCAD by tracing experimental images of FITC-dextran perfused vessels. Flow of medium was modeled using free and porous media flow and laminar flow physics, and the momentum transport modules were coupled with transport of diluted species physics as detailed previously [30] to simulate transport of oxygen, glucose, and insulin. The islets consumed oxygen and glucose, and secreted insulin with previously reported kinetic functions [31]. The PDMS was permeable to oxygen and media entering the device was in equilibrium with the atmospheric oxygen [32]. Glucose is introduced through the inlet and the insulin and glucose output was measured by using time integral of outlet amount over 10-minute intervals. The constants used in the model are given in table S3.

***Generating pseudoislets.*** Unsized and non-dithizone stained islets were recovered overnight in islet medium and then digested with Accutase (Thermo Fisher) for 12 min in a 37 °C water bath with regular agitation. After gentle trituration the Accutase was quenched with islet medium and cells were recovered by centrifugation. Islet cells (600 000/well) were seeded into Aggrewell 400 24-well plates (StemCell Technologies, Vancouver Canada), and mixed with EC cells at a 5:1 ratio. Pseudoislets were cultured for 7–14 d in long-term islet media (table S2). Pseudoislet function was measured by static GSIS at 14 d post-reaggregation. 24h prior to loading, 200k ECs were added into each Aggrewell and the plate was spun at 180 g for 2 min. to coat the surface of the pseudoislet. For generation of the islet-VMO, pseudoislets were co-loaded 7 d after reaggregation (10–20 islets *μ*l^−1^ hydrogel) as detailed earlier.

***Immune modeling.*** Single-donor peripheral blood mononuclear cells (PBMCs, 200 000/well, AllCells LLC, Alameda CA) were cultured for 5 days in round-bottom, ultra-low attachment 96-well plates (Corning) along with Interferon gamma (IFN-*γ*)-activated islets (10/well) and PBMC activation cocktail (table S1), in the presence or absence of major histocompatibility complex (MHC) class I and class II blocking antibodies, 10 mg ml^−1^ each (table S2). Resultant cell populations were tested for cytotoxic activity by incubation with donor-matched islets (10 islets/well) for two days. Supernatant was then collected for cytokine analysis by ELISA (Abcam, Cambridge MA). Whole islets were also incubated with Hoechst 33 342 and LIVE/DEAD Reduced Biohazard Cell Viability Kit (Thermo Fisher). Dead cells per islet were quantified using a custom MATLAB script to quantify total Dead^+^ cells as a percentage of total Hoechst^+^ cells (figure S4).

For perfusion through the islet-VMO, PBMCs were stained with 1 *μ*g ml^−1^ Cell Tracker Green (CMFDA) dye according to manufacturer’s protocol (Thermo Fisher Scientific) and then added to the input well (V_A_) at 5 × 10^5^ cells ml^−1^. Vessels were flushed with medium before PBMC quantification to remove non-adherent cells. PBMCs either in the vessels (adherent) or in the extravascular space (extravasated) were quantified. Extravasated PBMC within regions of interest defined as the outline of an islet (invasive), or within 100 mm of the islet (islet-adjacent) were counted. These regions of interest were then copied and pasted to areas devoid of islets and PBMC in these areas were quantified to give a value for background PBMC extravasation.

PBMC mediated cell killing was investigated through labeling of DNA strand breaks by TUNEL staining. Briefly, devices were stained utilizing the *In Situ* Cell Death Detection Kit (Sigma Aldrich 12 156 792 910) according to the manufacturers protocol and counterstained by 1 *μ*g ml^−1^ DAPI for 5 mins. Confocal image stacks of device-embedded islets were acquired on a Leica TCS SP8 confocal microscope.

***Statistical analysis.*** The Grubbs outlier test was used to determine statistical outliers within sample groups. Statistical significance (*p < 0.05, **p < 0.01, ***p < 0.001, ****p < 0.0001) was determined using either an unpaired, two-tailed Student’s *t*-test or ANOVA with Tukey post-hoc test, as appropriate, using GraphPad Prism 9 software (GraphPad Software, La Jolla CA).

## 3. Results

***Blood vessel formation in the islet-VMO platform.*** To generate an *ex vivo* endocrine pancreas model, we leveraged our VMO technology to create a 3D tissue with living, perfusable blood vessels that supply nutrients to the embedded islets. This platform consists of two microfluidic channels, C_1_ and C_2_ (figure 1(A)), flanking a central tissue chamber. To generate blood vessels, hydrogel containing EC and stromal cells is loaded from the side tunnels into the central cell chamber (figure 1(B)). To form the islet-VMO, islets are loaded together with these blood-vessel forming cells. A blood-substitute medium is supplied from four medium reservoirs, V_A_–V_D_, and is driven through the channels and into the central cell chamber by hydrostatic pressure flow through C_1_ (the functional arteriole), into the cell chamber, and out through C_2_ (the functional venule). Cells are initially fed by interstitial flow of medium through the hydrogel, and then by convective flow through vessels once they form (figure 1(B)).

To demonstrate proper vessel network formation, islet-VMO were perfused with 70 kDa FITC-dextran upon visual appearance of lumenized vessels, between 5 and 7 d. Vessel networks demonstrated preferential flow of dye through the vessel lumen and absence of fluorescent signal in the extravascular space immediately after dye perfusions (figure 1(C)). All islet-VMOs were perfused and vessel formation confirmed before future studies were conducted. *In vivo*, a feature of stable microvessels is the presence of pericytes that wrap around the vessels and promote a mature phenotype. As we have noted before [26, 33] a proportion of the stromal cells we add can differentiate into pericytes, and we see that also in the islet-VMO (figures 1(D) and (E)). Additionally, the presence of a laminin-rich basement membrane—a key hallmark of microvessels—is found proximal to the abluminal membrane of CD31^+^ blood vessels (figures 1(D)–(G)).

Lastly, to determine whether ECs from different sources form similar networks in the islet-VMO, we compared the vessel network-formation capacity of the ECFC-EC used up to this point with human umbilical vein EC (HUVEC). Mature networks were again perfused with 70 kDa FITC-dextran, and quantitated for total vessel length, number of branches, and internal diameter (figure S1). Both ECFC-EC and HUVECs yield networks of comparable morphology and similar distribution of vessel diameter (figure S1). While we chose to use ECFC-EC for our studies, both EC sources are suitable in the islet-VMO.

**Figure 2.**
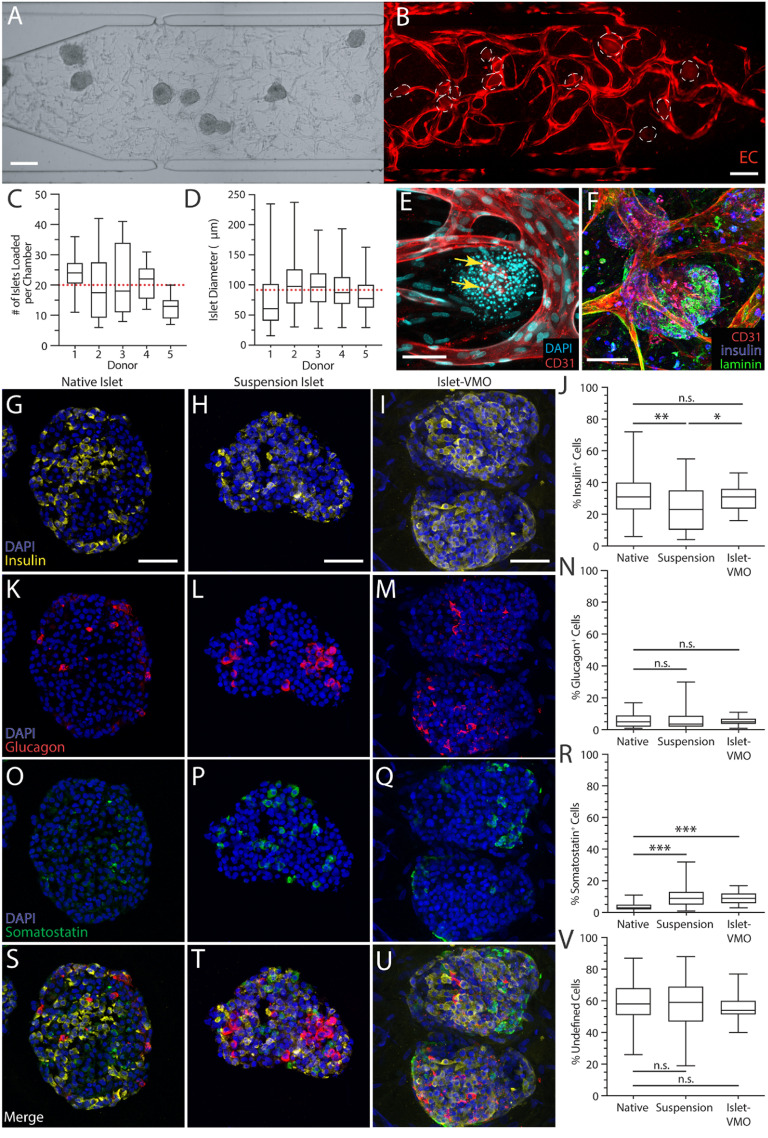
The islet-VMO platform supports islet-proximal vascularization and preserves islet cytoarchitecture. (A) Islets were loaded together with ECs and stromal cells such that even distribution of all populations are observed after loading. (B) Imaging of the same network after one week of maturation shows an intact vessel network (red, transduced ECs) around the embedded islets (dashed outline). Scale bar, 200 *μ*m. (C) The average number of islets was quantified per chamber across five donors, yielding an average of 20 islets loaded per chamber (red dashed line). (D) Quantification of average islet diameter across the same islet set shows an average islet diameter of 87.9 *μ*m (red dashed line) (*n* > 110 islets). Box and whisker plots represent median, 25th, and 75th percentiles (box) and min and max values (whiskers) for each data set. (E) Immunofluorescent staining for CD31-expressing endothelium (red) shows vessel formation immediately proximal to islets (DAPI, cyan). (F) Immunofluorescent staining of laminin (green) shows the presence of basement membrane surrounding islets (insulin, blue) and CD31+ endothelium (red). Scale bar, 75 mm. Immunofluorescent staining and quantification of (G)–(J) insulin^+^ β cells, (K)–(N) glucagon^+^ α cells, and (O)–(R) somatostatin^+^ δ cells was compared between cryosectioned native islets, cryosectioned islets maintained for one week in suspension culture, and *in situ* imaged islet-VMO-embedded islets (*n* > 45 islets across 8 donors). All images were acquired on a confocal microscope and represent maximal projections of the combined image stack. Scale bars, 50 mm. (S)–(U) Merged images of all staining and (V) quantitation of the unstained (non-endocrine) cells in each islet population.

***The islet-VMO platform supports islet vascularization and preserves islet cytoarchitecture.*** To match the dimensions of our microfluidic device we size-selected islets <200 *μ*m in diameter for loading with the EC and stromal cells. While islets of this size constitute the lower 50th percentile of islets in an average donor, evidence indicates that smaller islets (<125 *μ*m) contain more *β* cells, higher insulin content, and are more glucose responsive than larger islets (>150 *μ*m) from the same donor [34]. We found that islets were distributed evenly throughout the tissue chamber post-loading (figure 2(A)), which is a crucial step for ensuring uniform vessel formation (figure 2(B)). Across a random subset of donors, an average of 20 islets were loaded per chamber (figure 2(C)) with an average diameter of 88 *μ*m per islet (figure 2(D)).

We note that lumenized vessels formed immediately proximal to islets within 5–6 d post-loading, but did not penetrate them (figure 2(E)). Although CD31^+^ EC fragments are visible in some islets (presumably the surviving endogenous islet ECs), these cells do not elongate or connect with the outside vessels. Additionally, laminin staining demonstrates the presence of characteristic basement membrane surrounding islets in the islet-VMO, just as in the human pancreas [35] (figure 2(F)). Together, these data indicate that islets do not disrupt the surrounding vessels, nor do the vessels disrupt or penetrate the laminin basement membrane surrounding the islets [36].

To determine whether the cytoarchitecture of islets within the islet-VMO is maintained, we compared the proportion of insulin-, glucagon-, and somatostatin-expressing cells in native (freshly-isolated) islets, islets cultured for one week in suspension culture (‘suspension islets’), and islets cultured for one week in the islet-VMO. We found that suspension islets have fewer insulin^+^ cells compared to native islets, whereas insulin^+^ cells are maintained in the islet-VMO (figures 2(G)–(J)). Glucagon^+^ cell proportions are similar across all three islet populations (figures 2(K)–(N)), but the number of somatostatin^+^ cells is increased in both the suspension and islet-VMO islets, relative to native islets (figures 2(O)–(R)). In all islet populations, the level of undefined (non-endocrine) islet cells remains the same (figures 2(S)–(V)). Together, these data indicate that the islet-VMO maintains native islet cytoarchitecture and represents an improvement over standard islet suspension culture.

**Figure 3.**
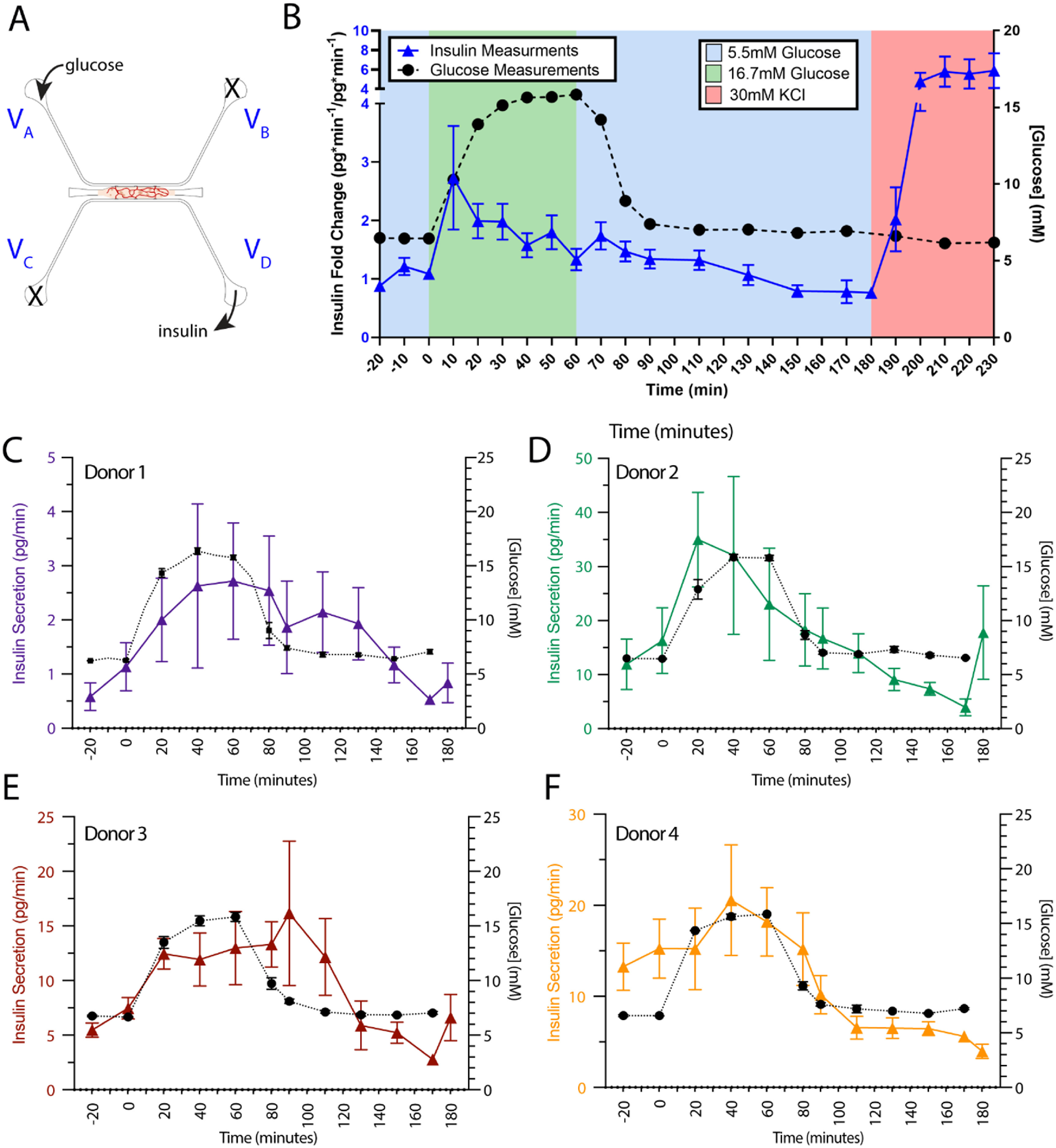
Glucose responsiveness of islet-VMO islets partially reflects *in vivo* glucose responsiveness. (A) To test islet response to glucose stimulation, medium supplemented with either basal (5.5 mM) or high (16.7 mM) glucose can be introduced through reservoir V_A_ to stimulate islets by perfusion through the vascular network. Insulin and glucose is measured from effluent collected at reservoir V_D_. Reservoirs V_B_ and V_C_ are blocked so that all glucose flows through the vessel network and secreted insulin is not diluted by cross-flow from V_C_. (B) Perfused glucose (black dashed line) and secreted insulin (blue line) are measured at 10 min intervals from collected effluent and plotted over time (*n* = 19 Islet-VMOs containing islets from 6 different donors). Error bars represent SEM. (C)–(F) Traces of measured glucose (black dashed line) and insulin (colored lines) from individual donors show similar secretion patterns despite different magnitudes of insulin secretion (note different *y*-axis scale) (*n* ⩾ 3 Islet-VMOs per donor).

***Glucose-responsiveness of islet-VMO islets reflects* in vivo *islet responses.*** To determine whether islet-VMO islets respond to glucose stimulation, we performed a GSIS assay by adding glucose-supplemented medium to reservoir V_A_ and collecting effluent from reservoir V_D_ (figure 3(A)). Reservoirs V_B_ and V_C_ were blocked to ensure all glucose flowed through the tissue chamber and that secreted insulin was not diluted by medium cross-flow in the venule channel. In response to perfusion with high glucose (16.7 mM) medium we observed gradual and near-simultaneous increases in both glucose and insulin in effluent collected from the platform (figure 3(B)). On average, Insulin release peaks 10 mins after addition of High glucose (10 min, 3.67 ± 1.872). Returning these platforms to 5 mM glucose medium results in a reduction to baseline insulin levels, demonstrating that these islets can reduce insulin secretion under low glucose conditions. Finally, KCl stimulation triggers a spike in measured insulin, demonstrating that the insulin secretory machinery remains fully intact in islet-VMO islets. Despite a consistent trend of glucose responsiveness across multiple islet donors, we found significant inter-donor variation in insulin secretion profiles. Responses can be slightly delayed (figure 3(C)), more rapid (figure 3(D)), peak later (figure 3(E)), or rise from a higher unstimulated baseline (figure 3(F)). Similar donor variation has been documented elsewhere, suggesting that donor islets fall into distinct functional subgroups [37]. As discussed later, the relatively small number of islets in each device may accentuate this heterogeneous insulin response.

**Figure 4.**
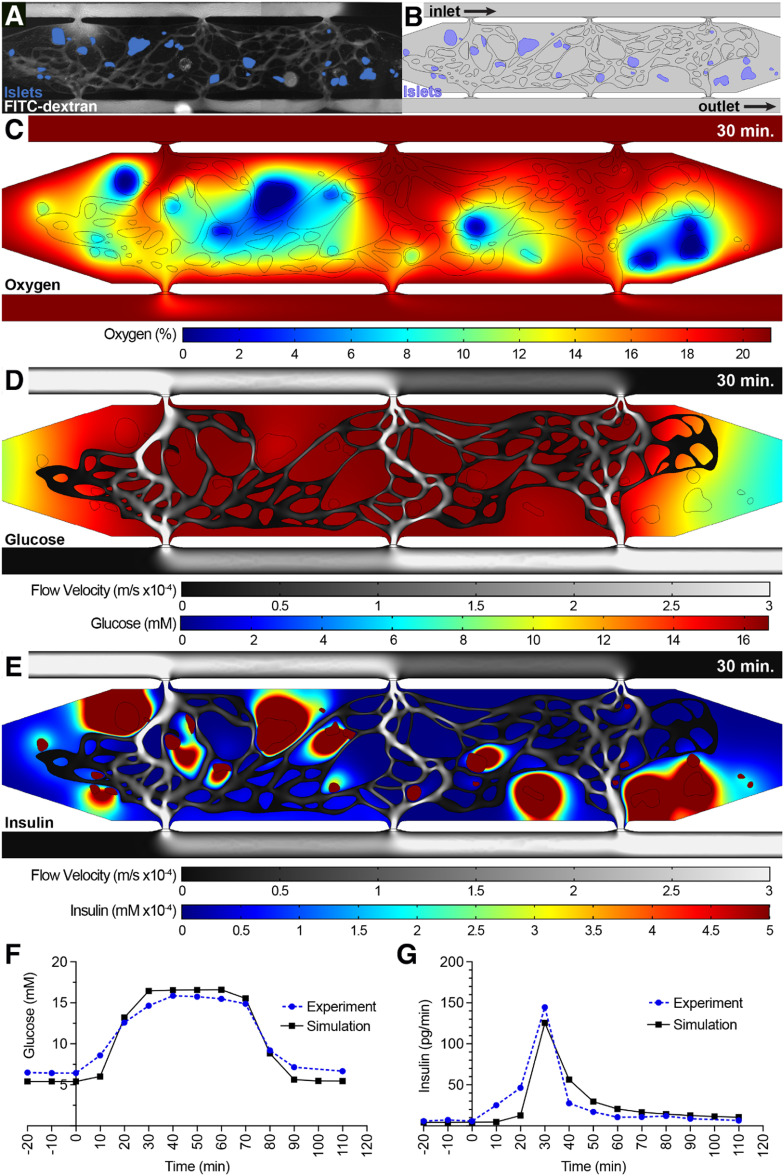
COMSOL modeling of islet function within the islet-VMO platform. (A) Islet-VMO platforms perfused were with FITC-dextran (gray) such that both the perfused vasculature and position of islets within the islet-VMO could be mapped. (B) For COMSOL modeling, vessels were converted to a skeletonized vessel network for COMSOL modeling (black outline) and islets localized to their mapped locations (blue). Glucose perfusion was modeled entering the model from the inlet with glucose and insulin measurements modeled at the outlet (equivalent to V_A_ and V_D_ in figure 3). Snapshots were acquired from the model at 30 min after high glucose perfusion showing (C) islet oxygen consumption (%, color scale), (D) medium velocity (m/s, gray scale) and glucose diffusion (mM, color scale), and (E) medium velocity (m/s, gray scale) and insulin secretion (mM, color scale). (F) Glucose perfusion and (G) insulin secretion traced over time in the COMSOL simulation model (black line) was compared to experimental values from the same islet-VMO (blue line).

***Mathematical modeling of islet function in the islet-VMO.*** To better understand the dynamics of glucose and insulin perfusion through the islet-VMO, we employed finite element modeling of actual islet-VMO networks using COMSOL MultiPhysics. To model networks, images of FITC-dextran perfused vessels (figure 4(A)) were traced and converted to a two-dimensional model with accurately sized islets positioned at the same locations as in the islet-VMO (figure 4(B)). Using the same hydrostatic pressure heads, glucose concentrations, and time intervals as those tested in the islet-VMO, the mathematical modeling allows estimation of spatial profiles of oxygen consumption, glucose diffusion, and insulin within the islet-VMO (figures 4(C)–(E)). As expected, we see high levels of oxygen consumption near islets (figure 4(C)), consistent with the high metabolic activity of human islets. Higher glucose concentrations are found closest to perfused vessels, as expected, whereas islets near the periphery receive comparatively less glucose due to their distance from the glucose source—the perfused vessels—and the glucose diffusion rate through the ECM (figure 4(D)). At least some insulin secretion is observed from all islets, however we note that secreted insulin pools in the vicinity of islets not bounded by a perfused vessel. To validate the mathematical model, we measured actual glucose and insulin concentrations sampled from port *V*_D_ (from one of the Donor 2 devices—aggregated data shown in figure 3(D)) and compared these to values determined by mathematical modeling of the same vascular network supplemented with islets of the same size and placement (figures 4(F)–(G)). We note that the sharp insulin secretion spike in the experimental data mirrored the response of islets from this donor set (Donor 2), which responded more rapidly than other sets (see figure 3(D)). Together, these data show that the simulation matches well with the experimental data.

**Figure 5.**
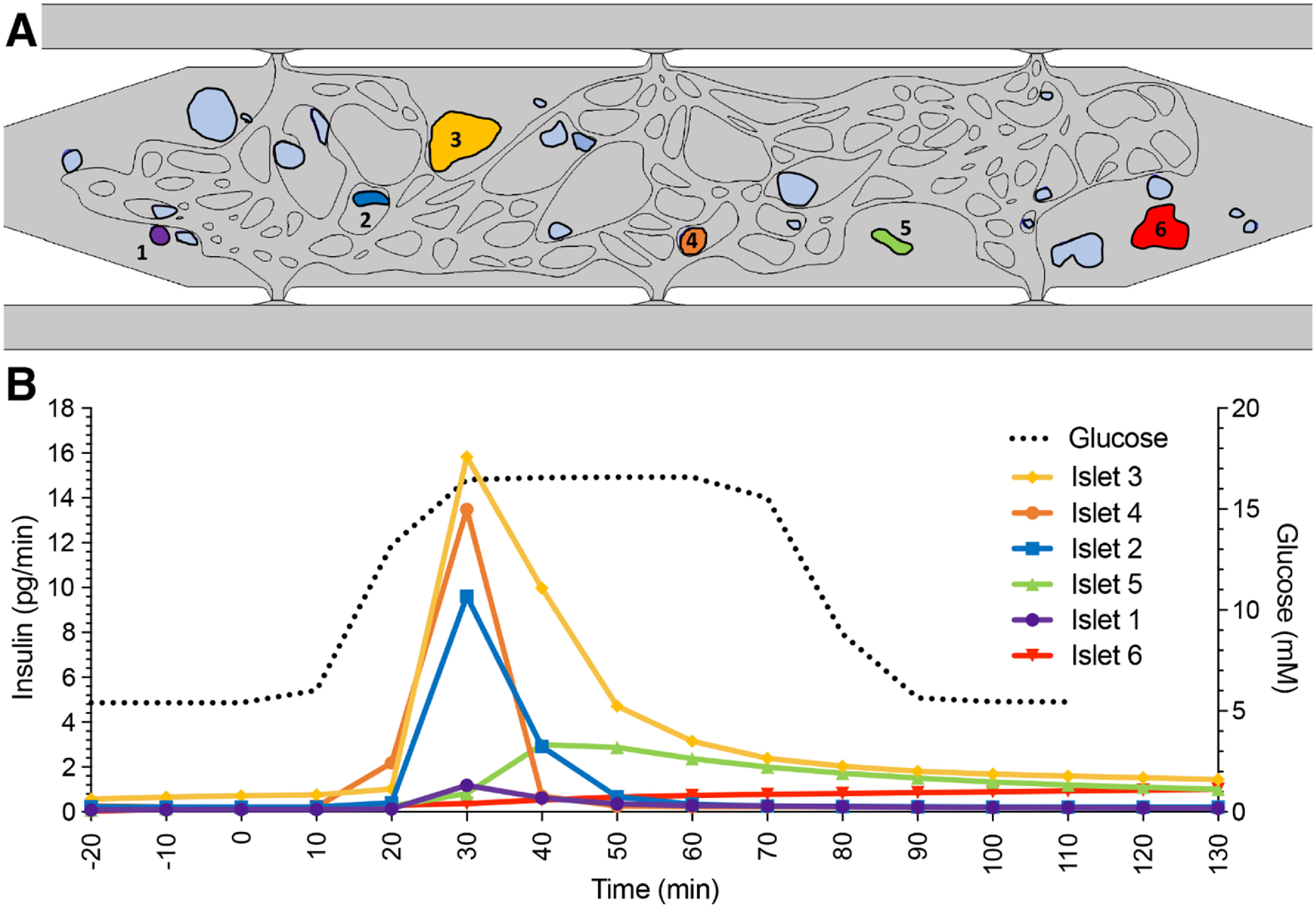
Individual islet function modeled within the islet-VMO. Using the COMSOL model described in figure 4, (A) a selection of six islets (annotated and colored) were selected for measuring individual islet function. (B) Insulin secretion profile (colored lines matched to islet color) for each islet was modeled over the course of low and high glucose perfusion (black dashed line), showing variable islet responses depending on location within the chamber and proximity to perfused blood vessels.

As noted above not all islets generate insulin at the same rate. To understand how individual islets contribute to overall insulin output, we mathematically shut down insulin secretion from all but one islet, and repeated this to examine islets individually (figure 5). Analyzed islets were selected based on: (a) their location within the chamber (either in the center [#2-5] or at the periphery [#1, #6]); and, (b) their proximity to perfused vessels (either close [#1-4] or distant [#5-6]) (figure 5(A)). Consistent with the hypothesis that perfused vessels allow for efficient transportation of islet-secreted insulin, islets closest to vessels (#2-4) demonstrate the highest release of insulin into the vessels as measured at the outlet V_D_ (figure 5(B)). However, islets at the periphery where vessels have lower perfusion (#1) contribute minimally to overall insulin output. In addition, islets distant from perfused vessels (#5, #6) also demonstrate both delayed and diminished insulin secretion, independent of their location within the chamber. To further demonstrate the importance of perfusable vessels in proximity to islets for improved glucose delivery and insulin clearance, we performed additional modeling on islet #3, in which a new vessel was added to the top side of the islet (figures S2(A)–(D)). Addition of the vessel dramatically reduces the extravascular accumulation of insulin surrounding the islet and increases insulin levels measured at the outlet (figures S2(E) and (F)). Together, these simulation data demonstrate the importance of vascular proximity, not only for the delivery of oxygen and glucose to islets, but also for the capture and distribution of insulin.

**Figure 6.**
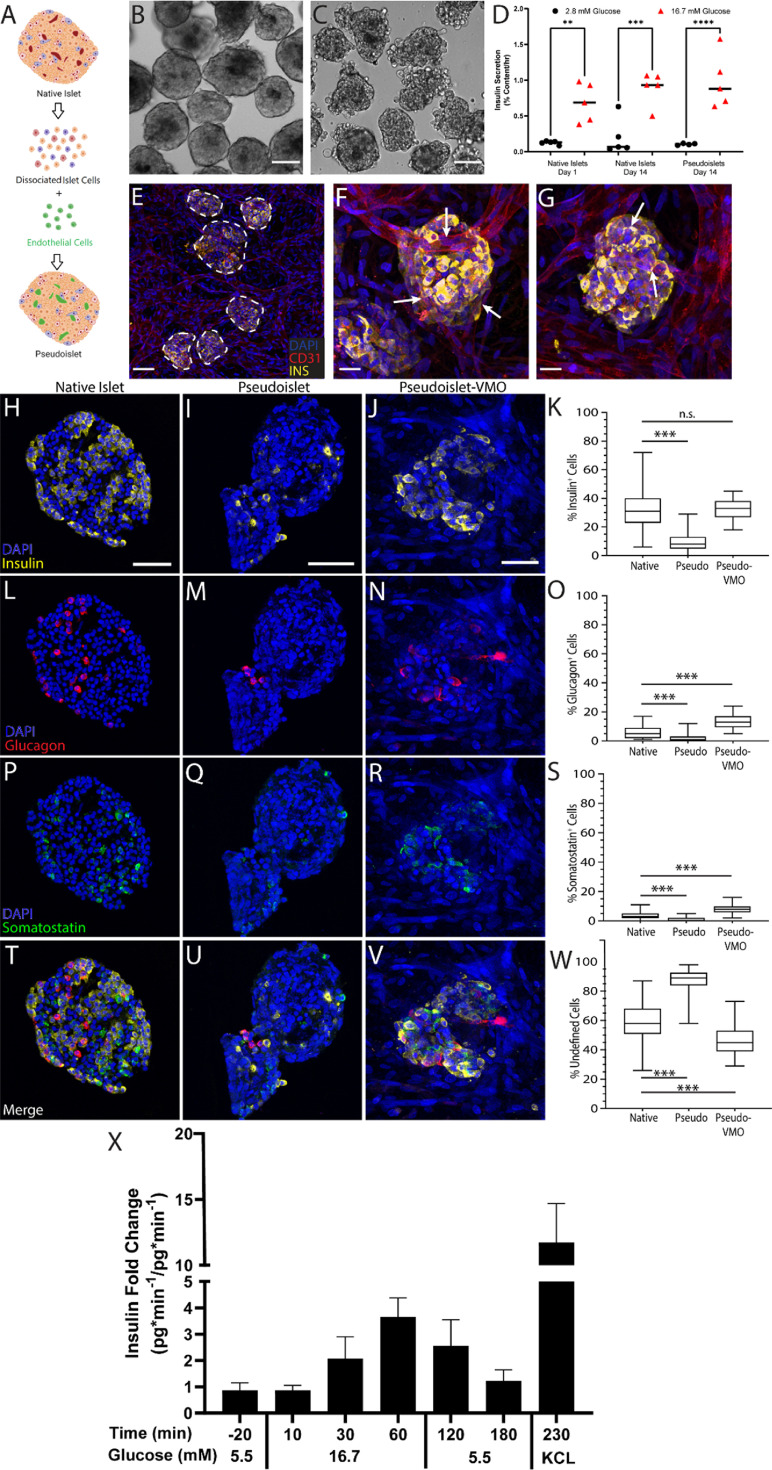
Intra-islet vasculature is enhanced in pseudoislets embedded within the islet-VMO. (A) To generate reconstituted islets, native islets were dissociated and reconstituted either as islet cells alone (reaggregated islets) or together with ECs (pseudoislets). Created with BioRender.com. (B)–(C) Islet morphology was compared by phase contrast microscopy two days post-reconstitution between (B) native islets and (C) pseudoislets. Scale bar, 100 *μ*m. (D) Static glucose-stimulated insulin secretion (GSIS) was measured for each islet population under unstimulated (2.8 mM glucose) and stimulated (16.7 mM) conditions (two-way ANOVA, significance: ns, not significant; **p < 0.01; ***p < 0.001). (E) Pseudo-islets were loaded together with EC and stromal cells and vessel networks allowed to form. ECs (CD31^+^, red) form an interconnected network that surrounds and penetrates the pseudoislets (DAPI, cyan, dashed outline). Scale bar, 100 mm. (F)–(G) CD31 staining shows vessels penetrating pseudo-islets (white arrows). Scale bar, 25 *μ*m. Immunofluorescent staining and quantification of native islets, pseudoislets after reconstitution, and pseudoislets maintained in the islet VMO for one week (pseudo-VMO) for (H)–(K) insulin^+^, (L)–(O) glucagon^+^, and (P)–(S) somatostatin^+^ cells. Scale bar, 50 *μ*m. (Student’s *t*-test, n.s., not significant; ***p < 0.0001). (T)–(W) Merged images of staining in all islet types and (Y) quantitation of undefined (non-endocrine) islet cells. (X) Secreted insulin at listed timepoints collected over a 10 minute interval (*n* = 7 Islet-VMOs containing islets from 2 different donors). Error bars represent SEM.

***Pseudoislets support intra-islet vasculature.*** As noted above, intact cadaveric islets often retain small CD31+ vessel fragments but these fragments do not anastomose with the vasculature in the VMO. We therefore looked to see whether vasculature could be incorporated into dissociated and then re-aggregated islets, i.e. pseudoislets. We dissociated native islets and reconstituted these with ECs (figure 6(A)), and found that, in contrast to isolated native islets (figure 6(B)), the pseudoislets have less well-defined boundaries and are generally more loosely packed (figure 6(C)). We then assessed viability of the pseudoislets by performing a time course of static GSIS assays and found that a full return to glucose sensitivity required 14 d (data not shown). A comparison of native islets 1 d post-delivery and 14 d post-delivery, with day 14 pseudoislets found that pseudoislets have a comparable insulin release proportional to content as native islets (figure 6(D)). However, the absolute insulin content and release of these pseudoislets is decreased relative to 14 d donor-matched native islets (figure S3).

To determine whether incorporation of ECs into the pseudoislets facilitates improved intra-islet vasculature in the islet-VMO platform, pseudoislets were co-loaded along with additional EC and stromal cells into the platform seven days post-reconstitution. We found blood vessels that both penetrated the pseudoislets and closely associated with the surface (figures 6(E)–(G)). To examine endocrine cell ratios following reconstitution, we performed immunofluorescent staining of pseudoislets 48 h after reaggregation and after one week in the islet-VMO and compared these to native islets. In the islet-VMO platform, pseudoislets showed a similar number of insulin^+^ cells as native islets, whereas these cells were considerably diminished in number in the pseudoislets cultured in suspension (figures 6(H)–(K)). Interestingly, while the number of glucagon^+^ and somatostatin^+^ cells was also decreased in suspension pseudoislets compared to native islets, both populations were significantly enriched in pseudoislets in the islet-VMO (figures 6(l)–(O) and (P)–(S)). Consistent with the degraded cytoarchitecture of suspension islets they had a greatly increased number of undefined (non-endocrine) cells compared to the other groups (figures 6(T)–(W)).

We next performed GSIS assays on day 14 pseudoislets in the islet-VMO platform, and consistent with our data using intact islets found a robust response to glucose challenge and KCl stimulation (figure 6(X)). Interestingly, we noted a delay in insulin release in response to high glucose with the pseudoislets compared to intact islets, although the maximum levels achieved were comparable. This may reflect an imbalance in the numbers of *α, δ* and *β* cells, which could disrupt the complex cross-regulatory pathways that normally operate to fine-tune insulin release. This idea is consistent with previous work demonstrating that both *α* and *δ* cells can regulate *β* cell insulin release [38]. Consistent with our static GSIS studies, freshly reaggregated islets in the islet-VMO demonstrated little to no insulin secretion despite comparable insulin content (data not shown).

**Figure 7.**
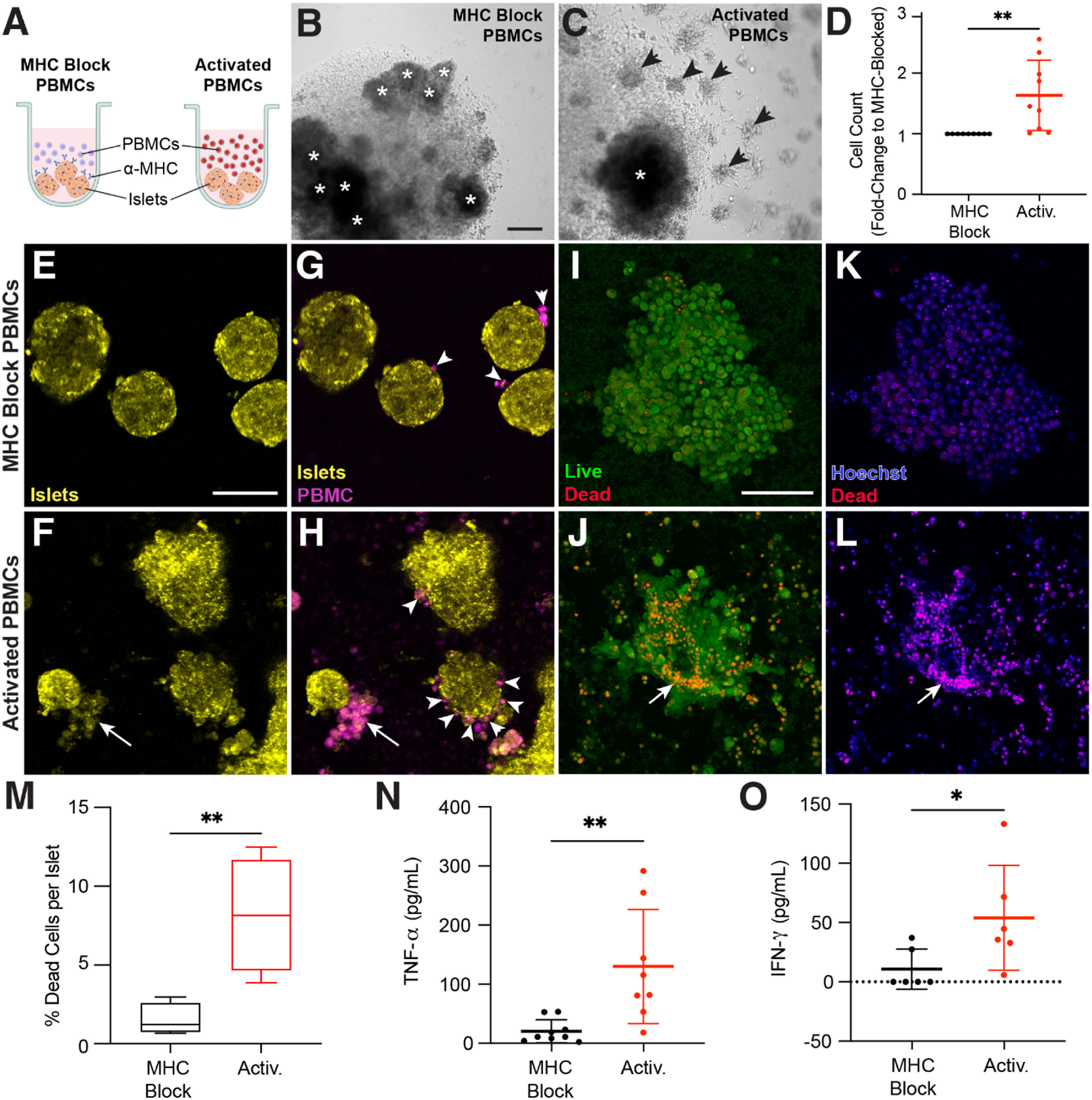
PBMCs are activated against donor islets. (A) MHC blocked and activated PBMCs were generated by incubating PBMCs with donor islets and IL-2 + IFN-g (activation cocktail) in round-bottom 96-well plates. MHC blocked conditions also contained antibodies against both MHC Class I and II. Schematic created with BioRender.com. After 5 d under these conditions, PBMCs cultured in the (B) presence or (C) absence of MHC-blocking antibodies demonstrates clonal expansion (activation) only in the absence of MHC-blocking antibodies. Islets are annotated by asterisks (*) and grape-like clusters annotated by arrowheads. Scale bar, 200 *μ*m. (D) Quantification of activated PBMCs (red) as a fold-change relative to MHC blocked PBMCs (black) after five days of activation. These MHC blocked and activated PBMCs were isolated and incubated with islets from the same donor for 48 h. (E)–(H) Islets (yellow) and PBMCs (magenta) were stained with different CellTracker dyes and imaged for interactions between the two after 48 h incubation in a chamber slide. Islets exposed to activated but not MHC blocked PBMCs show morphological damage Scale bar, 50 *μ*m (E), (F). (G) MHC blocked PBMCs show minimal affinity for islets (white arrowheads) whereas (H) activated PBMCs show increased adhesion to islets and, in some cases, destruction of islet tissue (white arrows). (I)–(L) Islets from the same conditions were stained with live/dead stain. (I), (J) Morphological damage is evident in islets exposed to activated but not MHC blocked PBMCs (arrow indicates islet damage). (K), (L) Dead cell staining (red) shows increased dead cells in islets exposed to activated but not MHC-blocked PBMCs. (M) Quantification of dead cell labeling (red) versus Hoechst (nuclei, blue) staining in islets exposed to MHC blocked (black) or activated (red) PBMCs (Student’s t-test, **p < 0.01) (*n* = 30 islets across 4 donors). Supernatant was collected from these co-incubations and measured for secreted (N) TNF-α and (O) IFN-γ relative from MHC blocked (black) and activated (red) PBMCs. Statistical significance determined by Student’s t-test (significance: *p < 0.05, **p < 0.01).

***Introduction of islet-activated immune cells as a model for islet-immune cell interactions.*** To assess the utility of our platform for investigating immune cell interactions with islets, we developed a non-autologous system for proof-of-concept studies. To enrich for alloreactive T cells we cultured non-MHC matched PBMCs with freshly-isolated islets either alone, or in the presence of blocking antibodies to MHC class I and II (figure 7(A)). IFN-*γ* was added to increase MHC molecule expression and IL-2 was added to drive proliferation of any T cells that became activated by allogeneic MHC. As expected, a sub-population of the T cells in the PBMC population did indeed have T cell receptors that cross-reacted with the (foreign) islet MHC, leading to activation and proliferation (as seen by grape-like clusters of cells), and this was blocked by the anti-MHC antibodies (figures 7(B) and (C)). Proliferation was confirmed by cell counting (figure 7(D)). We refer to these two populations as ‘Activated’ and ‘MHC-blocked’ cells.

To determine the killing capacity of the two cell populations, activated and MHC-blocked cells were labeled with CellTracker Green and then incubated with CellTracker Red-stained whole islets from the same donor that was used for the initial stimulation. Live cell imaging after two days shows morphological damage to islets exposed to activated immune cells, but not those incubated with MHC-blocked cells (figures 7(E) and (F)). Moreover, MHC-blocked cells infrequently interact with islets whereas activated cells regularly surround islets (figures 7(G) and (H)). Examination of islets with live/dead staining shows that islets exposed to activated, but not MHC-blocked cells experience morphological damage (figures 7(I) and (J)). Islets exposed to activated cells also have a higher percentage of dead cells than those exposed to MHC blocked cells (figures 7(K)–(M)). ELISA analysis of the supernatant from these assays also shows increased secretion of the inflammatory cytokines TNF-*α* and IFN-*γ* from activated cells interacting with the islets (figures 7(N) and (O)).

**Figure 8.**
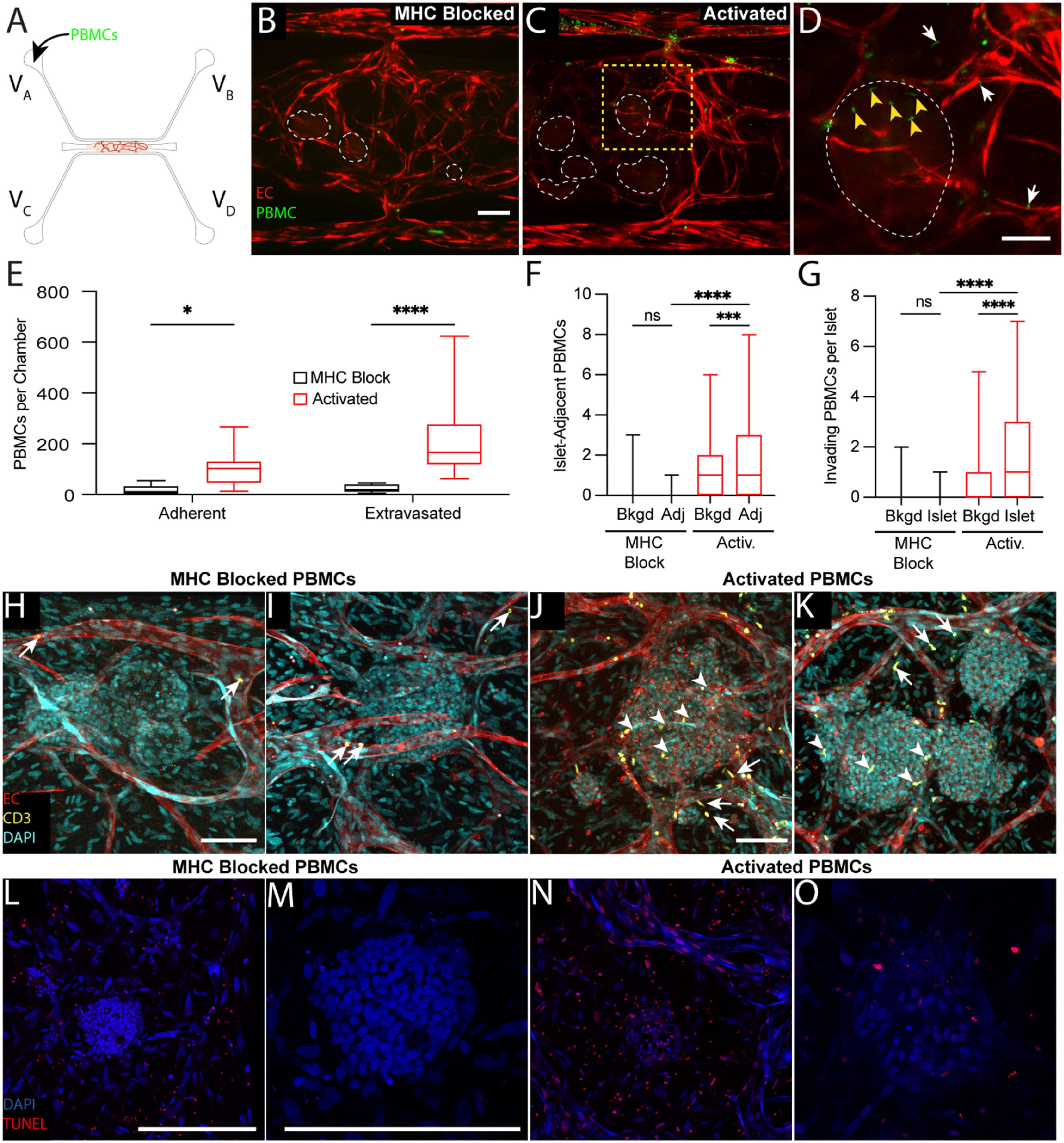
Immune cell and islet interactions are modeled within the islet-VMO. (A) CellTracker+ MHC-blocked or activated PBMCs were added to reservoir VA and allowed to perfuse though islet-VMOs containing islets from the same donor for up to 48 h. (B) MHC-blocked PBMCs (green) demonstrate minimal adhesion or extravasation from vessels (fluorophore-transduced ECs, red) as compared to (C) activated PBMCs (islets, dashed outline). Scale bar = 200 *μ*m. (D) Inset from (C) (yellow dashed box) shows increased migration of activated PBMCs out of the vasculature towards the islet (white arrows) with multiple PBMCs co-localizing within the islet itself (yellow arrowheads). (E) Quantification of PBMC staining shows increased adhesion and extravasation of activated but not MHC-blocked PBMCS across multiple islet donors (two-way ANOVA, *p < 0.05, ****p < 0.0001) (*n* = 14 Islet-VMOs across 4 islet donors). (F) The number of PBMCs within 100 *μ*m of an islet was quantified (Adj) and compared to both background, non-islet regions (Bkgd) and MHC-blocked PBMCs (one-way ANOVA, ***p < 0.001, ****p < 0.0001) (*n* > 65 islets across 4 islet donors). (G) The number of PBMCs present within islets (islet) was quantified and compared to PBMC counts in background, non-islet regions (Bkgd) and MHC-blocked PBMCs. (one-way ANOVA, ***p < 0.001, ****p < 0.0001 by) (*n* > 65 islets across 4 islet donors). (H), (I) Confocal imaging of fixed and stained islet-VMOs shows minimal adhesion (arrows) of perfused with MHC-blocked CD3+ T cells (green) to the vessels (CD31+, red; DAPI, cyan). (J), (K) Immunofluorescent staining of islet-VMOs perfused with activated PBMCs shows increased extravasation (white arrows) and islet (DAPI, cyan) invasion (yellow arrowheads) of CD3+ T cells. (L,M) TUNEL staining of islet-VMOs perfused with MHC-blocked-PBMCs shows little to no cell death in the islets. Scale bars, 100 *μ*m. (N), (O) TUNEL staining of islet-VMOs perfused with activated PBMCs shows evidence of cell death in the islet. Scale bars, 100 *μ*m.

Having determined that islet-reactive lymphocytes can be generated in response to allogeneic islets, we next sought to determine whether these cells can traffic to islets within the islet-VMO. CellTracker-stained populations of either MHC-blocked or activated PBMC were added to reservoir V_A_ and perfused through platforms containing islets from the same donor used for priming (figure 8(A)). After 48 h of perfusion we noted robust extravasation of activated cells whereas we saw very few MHC-blocked cells entering the tissue (figures 8(B) and (C), S5(A) and (B)). Many of the extravasated cells migrated towards the islets, with some also visible within the islet structure itself (figure 8(D)). Indeed, activated PBMCs demonstrated significantly higher rates of adhesion and extravasation (figure 8(E)). To determine if activated cells preferentially traffic to (or are retained in) islets, the number of immune cells within 100 *μ*m of an islet was quantified (Adjacent—Adj) and compared to control (figure 8(F)). To control for background (Bkgd) trafficking, a similar-sized area was used to quantify immune cells in non-islet regions of the same chamber. MHC-blocked cells showed no preference for islets compared to background regions (figure 8(F)). In sharp contrast, immune cells that had been pre-activated against islet MHC showed a strong and significant bias toward islet localization (figure 8(F)). To determine if activated PBMCs also invade islets, the number of immune cells within an islet was quantified and compared to both background (as described above) and MHC-blocked cells (figure 8(G)). Again, compared to the control cells, the activated cells were strongly biased toward islet invasion.

To confirm that it is indeed T cells invading the islets we stained tissues for CD3. We found very few CD3^+^ cells in tissues perfused with control (MHC-blocked) PBMC (figures 8(H) and (I)), whereas these cells were numerous both in and around islets in tissues perfused with activated PBMC (figures 8(J) and (K)). Thus, T cells with specificity (allogeneic) for islets can be introduced through the vasculature of the islet-VMO platform where they can extravasate and migrate into islets. To confirm extravasated PBMCs were capable of initiating cell death, TUNEL staining for fragmented DNA strands was used as an indirect visualization of the initiating steps for apoptosis. We noted numerous TUNEL-positive cells in islets perfused with Activated PBMCs, whereas there was little to no staining in islets perfused with MHC-blocked PBMCs (figures 8(M) and (O)). In both cases we did observe some positive staining outside of the islets (figures 8(l) and (N)). Thus, in aggregate or data show that in the islet-VMO platform activated T cells can enter islets through the vasculature and initiate cellular damage.

## 4. Discussion

Here, we describe a novel, biomimetic approach that models important aspects of the native islet environment while also preserving islet function *ex vivo*. Specifically, the islet-VMO uniquely incorporates: (a) a 3D microenvironment comprised of ECM and human stromal cells mimicking the native pancreas; (b) perfusable human blood vessels that transport glucose and insulin to and from islets; and, (c) the capacity to deliver immune cells via this vasculature. Importantly, the islets maintain glucose responsiveness for at least a week in the platform. Others have explored some of these strategies for improving human islet health, including recapitulating the surrounding ECM [39, 40], physiologically-relevant interstitial flow [41–43], and co-culture with ECs [44]. The islet-VMO, however, incorporates all of these features with the addition of a perfusable human vasculature, resulting in a uniquely powerful tool capable of investigating physiologically-relevant islet functions and immune interactions.

The kinetics of insulin secretion we see with the islet-VMO mirror those seen in healthy human subjects (figure 3) but differ somewhat from standard perifusion assays. In perifusions, islets exhibit strongly biphasic GSIS characterized by a brief, high-amplitude first phase, followed by a lower, sustained second phase [45]. In contrast, in the islet-VMO we see a more gradual slope of first-phase insulin release and the absence of a distinct second phase. Previous work has also shown the lack of a second-phase response when islets are encapsulated in a hydrogel [46], consistent with our findings. Our COMSOL modeling suggests that the islet response is determined both by location within the chamber and proximity to perfused vessels. Critically, the insulin secretion dynamics demonstrated in the islet-VMO match well with human *in vivo* insulin secretion (figure 3), which is also characterized by a gradual accumulation of circulating insulin without an apparent second phase of insulin release [47]. Also noted was the significant donor-to-donor variation. By bench-marking islet-VMO insulin secretion to the initial starvation phase, we found on average that the islet-VMO shows a 3-fold change in insulin secretion in response to a step increase of glucose concentration from 5 to 16.8 mM.

In this study we found that individual islet-VMO insulin traces demonstrated both intra- and inter- donor variation in both the magnitude of insulin secretion and in glucose response time. Islet variation has long been a problem in the field, only addressed through large sample sizes and/or aggregate testing of large numbers of islets. Recently, Granlund *et al* estimated that 400 islets per condition are required in order to adequately represent human variation [48]. Furthermore, many conventional assays of islet function require the destruction of the islets, precluding longitudinal studies of disease relevant perturbations in an *in vitro* setting. Recently published techniques have used microfluidic-based devices to allow for recovery of single islets after perfusion studies, however these are non-trivial in design and operation [21]. Although the islet-VMO demonstrates the same islet-islet variation, many of the studies presented here are not end-point assays, allowing for controlled experiments in disease relevant conditions. For example, device GSIS can be conducted before and after perfusion of PBMCs, in order to understand the effect of T cell-mediated damage of islets. These data demonstrate that islets within the islet-VMO respond to glucose stimulation, recapitulate islet donor-to-donor variation, and mimic the linked changes in glucose and insulin levels observed in the human body.

To establish the usefulness of the islet-VMO for modeling invasion of pancreatic islets by circulating immune cells, we conducted a proof-of-concept allogenic PBMC perfusion experiment. PBMCs co-cultured for 5 days in the presence of IL-2 and IFN-*γ* demonstrated a ready ability to adhere and extravasate upon perfusion through the device. Further, CD3 and TUNEL staining in devices perfused with activated PBMCs confirmed T cell migration to encapsulated islets and the presence of dying cells. MHC-blocking antibodies were not included with the perfusion of the MHC-blocked T cell control group, therefore it was unsurprising to see wide spread TUNEL staining throughout the device due to EC/PBMC HLA mismatch. However, there was a notable lack of TUNEL staining in islets encapsulated in devices perfused the MHC-blocked PBMC group, consistent with CellTracker dyes and immunofluorescent staining which demonstrated few T Cells had migrated into the islets in the MHC-blocked group. Taken together, we demonstrated that under appropriate stimuli, T Cells retain their ability to adhere to perfusable vasculature, extravasate into the extravascular space, and initiate cell apoptosis.

Diabetes is a complex disease characterized by both molecular and immunological triggers that drive disease pathogenesis. Interaction between immune cells and islets is important in both forms of diabetes, wherein T1D is driven by autoimmune reactivity of T cells against *β* cells [49] while T2D is characterized by *β* cell inflammation and activation of tissue resident and circulating macrophages [8, 10, 50]. In this study, we have demonstrated the feasibility of using the islet-VMO to understand these immune cell interactions. The model recapitulates physiological immune cell delivery through vessels, trafficking (extravasation), and islet invasion. While these processes can be modeled in mice, critically, the islet-VMO only utilizes human cells in a physiologically-relevant tissue and immune environment, thereby improving the correlative power of discoveries for patients. However, there are some important limitations to this model that should be noted. First, this model utilizes isolated islets from cadaveric tissue. We have previously noted that this isolation process disrupts the local ECM and vasculature. While this work demonstrates vasculature that associated with islets, there is little evidence of microvessel formation that would recapitulate islet geometry that is noted *in vivo*. Further, *β* cells exhibit a polar orientation in relation to local vasculature that impact glucose sensitivity and insulin release [51]. It is not clear that this cellular layout is recapitulated by pseudoislets, at least in the time-frame studied, possibly accounting for the variable glucose sensitivity exhibited between each device. Reliance on cadaveric derived pancreatic islets represents a significant cost and biological hurdle to adoption of this model. Second, T1D is a complex autoimmune disorder involving many different types of immune cells. The model described here only investigated one cell type, T Cells, and their ability to migrate to islets. Other cell types, particularly antigen-presenting cells and tissue resident macrophages will have to be investigated and incorporated into the model to recapitulate other aspects of T1D. Finally, T1D is an autoimmune disorder and the various cell types used in this study were derived from different donors. Further studies using this model to investigate T1D will need to rely on human leukocyte antigen matching or alternative tissue sources to derive an autologous model.

While the islet-VMO is suitable in its current form to explore questions related to inflammatory cytokines and autoantigen release, other questions on T1D progression—namely, how genetic polymorphisms or environmental factors alter T cell and *β* cells interactions—are best answered in a completely autologous system. We and others have achieved several milestones towards generating such a platform. First, recent work demonstrates the feasibility of deriving all of the relevant cell populations from iPSCs, including islet cells [52, 53], ECs [54], and stromal cells that can be paired with patient-matched immune cells. Second, in this work we have demonstrated an approach to generate pseudoislets from dissociated cell populations and incorporate these pseudoislets within a vascularized islet-VMO. We note that the level of insulin secretion from pseudoislets in the islet-VMO and the tight regulation of insulin release do appear somewhat disrupted relative to intact islets, potentially due to an imbalance of endocrine cell types and ongoing remodeling of the microenvironment. Future studies will investigate these possibilities. We anticipate that the pseudoislet approach will ultimately allow incorporation of several relevant iPSC-derived cell types leading to a fully autologous system. Importantly, β cells can be generated from iPSCs derived from T1D patients [55], enabling incorporation of *β* cells with susceptible genetic backgrounds into the platform for testing subsequent triggers of disease progression. Lastly, a similar but simpler 2D approach using iPSC-derived *β* cells and patient-matched PBMCs from both T1D patients and healthy volunteers has demonstrated the feasibility of using these cells to model relevant cell-cell interactions [56]. We anticipate that use of the islet-VMO will further broaden our understanding of human islet biology during T1D pathogenesis.

## Data Availability

All data that support the findings of this study are included within the article (and any supplementary files).
